# Dual Strategies of Metal Preintercalation and In Situ Electrochemical Oxidization Operating on MXene for Enhancement of Ion/Electron Transfer and Zinc‐Ion Storage Capacity in Aqueous Zinc‐Ion Batteries

**DOI:** 10.1002/advs.202206860

**Published:** 2023-01-16

**Authors:** Zhonglin Li, Yifan Wei, Yongyao Liu, Shuai Yan, Mingyan Wu

**Affiliations:** ^1^ State Key Laboratory of Structural Chemistry Fujian Institute of Research on the Structure of Matter Chinese Academy of Sciences Fuzhou 350002 P. R. China; ^2^ University of Chinese Academy of Sciences Beijing 100049 P. R. China; ^3^ College of Chemistry Fuzhou University Fuzhou 350108 P. R. China

**Keywords:** 2D materials, aqueous zinc batteries, electrochemical oxidization, metal preintercalation, V_2_CT*_x_* MXene

## Abstract

As an emerging two‐dimensional material, MXenes exhibit enormous potentials in the fields of energy storage and conversion, due to their superior conductivity, effective surface chemistry, accordion‐like layered structure, and numerous ordered nanochannels. However, interlayer accumulation and chemical sluggishness of structural elements have hampered the demonstration of the superiorities of MXenes. By metal preintercalation and in situ electrochemical oxidization strategies on V_2_CT*_x_*, MXene has enlarged its interplanar spacing and excited the outermost vanadium atoms to achieve frequent transfer and high storage capacity of Zn ions in aqueous zinc‐ion batteries (ZIBs). Benefiting from the synergistic effects of these strategies, the resulting VO*_x_*/Mn–V_2_C electrode exhibits the high capacity of 530 mA h g^−1^ at 0.1 A g^−1^, together with a remarkable energy density of 415 W h kg^−1^ and a power density of 5500 W kg^−1^. Impressively, the electrode delivers excellent cycling stability with Coulombic efficiency of nearly 100% in 2000 cycles at 5 A g^−1^. The satisfactory electrochemical performances bear comparison with those in reported vanadium‐based and MXene‐based aqueous ZIBs. This work provides a new methodology for safe preparation of outstanding vanadium‐based electrodes and extends the applications of MXenes in the energy storage field.

## Graphical abstract

Dual strategies of metal preintercalation and in situ electrochemical oxidization are developed for resolving interlayer accumulation and structural‐element chemical sluggishness of V_2_CT*_x_* MXene. Benefiting from the enlarged interplanar spacing of MXene and the outermost raised vanadium valence, the resulting VO*_x_*/Mn–V_2_C electrode exhibits high specific capacity, high energy density, and longevity in aqueous zinc‐ion batteries.

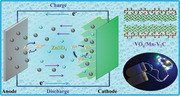

## 1 Introduction

In view of the ever‐increasing energy needs and environmental pollution associated with the combustion of fossil fuel, there is an urgent need to develop high energy, high security, and sustained electrochemical energy‐storage systems.^[^ ^1^, ^2^ ^]^ Rechargeable aqueous Zn‐ion batteries (ZIBs) have attracted increasing attention due to their prominent advantages of safety, high energy density, low expense, and reduced environmental costs.^[^ ^3^, ^4^, ^5^, ^6^ ^]^ Moreover, the aqueous electrolytes used in ZIBs possess competitive ion‐transfer ability and could cancel the flammable issues caused by organic electrolytes. Consequently, aqueous ZIBs are regarded as highly promising alternative energy‐storage systems for future applications. However, it is a still formidable challenge how to design a robust and high‐efficiency cathode material which could advance the commercialization of aqueous ZIBs.^[^ ^7^, ^8^, ^9^ ^]^

Recently, many kinds of materials have been designed to serve as the cathodes of aqueous ZIBs. These include vanadium‐based oxides,^[^ ^10^, ^11^, ^12^, ^13^ ^]^ manganese oxides,^[^ ^14^, ^15^, ^16^, ^17^ ^]^ organic molecules and polymers,^[^ ^18^, ^19^, ^20^, ^21^, ^22^ ^]^ Prussian blue analogs,^[^ ^23^, ^24^ ^]^ and other intercalation compounds.^[^ ^25^, ^26^, ^27^ ^]^ Among these, vanadium‐based oxides with many molecular formulas and crystal structures, commonly exhibit the high theoretical specific capacity and excellent cycling stability, owing to their multiple valence states and opened‐framework crystal structure.^[^ ^5^, ^28^, ^29^, ^30^ ^]^ However, insertion/extraction of Zn^2+^ in its crystal structure often results in intrinsic sluggish kinetics, irreversible phase transitions, and even structural collapse, leading to inferior electrochemical performances.^[^ ^11^, ^31^ ^]^ In addition, most vanadium‐based cathodes, especially those containing vanadium oxides, exhibit low electronic and ionic conductivity,^[^ ^32^, ^33^ ^]^ which hampers their Zn^2+^ storage capability. Although great attempts have been made to fabricate the vanadium‐based cathode materials for aqueous ZIBs, rational design of such materials is still in its infancy.^[^ ^11^ ^]^ Therefore, it is necessary to develop an applicable methodology for the preparation of the feasible vanadium‐based cathode materials with stable phase components and high electronic/ionic conductivities to improve the capacity and cycling life of ZIBs.^[^ ^29^, ^34^ ^]^

Over the past decade, MXenes have grown into key versatile 2D materials that have been widely applied in photodetectors,^[^ ^35^ ^]^ pollutant degradation,^[^ ^36^ ^]^ sensors,^[^ ^37^ ^]^ and energy storage and conversion.^[^ ^38^, ^39^, ^40^ ^]^ Equipped with nanolaminate microstructure, superior conductivity, and adequate surface chemistry,^[^ ^41^ ^]^ MXenes provide a great opportunity for fabrication of high‐performance ZIBs. As a member of MXene families, V_2_CT*_x_* possesses an accordion‐like 2D layered structure and numerous ordered nanochannels, which would be beneficial to electron transport and could provide adequate active sites for the insertion/extraction of Zn^2+^ ions.^[^ ^42^ ^]^ Unfortunately, pure V_2_CT*_x_* MXene delivers unsatisfactory electrochemical performances in aqueous ZIBs. It is speculated that the poor redox reactivity of V_2_CT*_x_* can be ascribed to its low valence V atoms and its shrinkable layer structure,^[^ ^15^, ^43^, ^44^ ^]^ which prohibits it from taking part in multielectron redox reactions and accelerating charge transfer during the charge/discharge process. Considering the classical multielectron transfer potential of vanadium, it could be possible to promote the Zn‐ion storage capability of V_2_CT*_x_* by raising the valence state of the vanadium atoms in V_2_CT*_x_*. Preferably, if the conductive 2D layered structure of V_2_CT*_x_* is deliberately preserved while the interplanar spacing of V_2_CT*_x_* is enlarged, high and fast Zn^2+^ storage capacities could be easily achieved in theory.

Herein, metal preintercalation into interlaminar spaces and the in situ phase transformation induced by electrochemical oxidization have been developed and have been found to enhance ion/electron transfer and the zinc‐ion storage capacity of MXene. Manganese ions intercalated into V_2_C (Mn–V_2_C) could enlarge the interplanar spacing of MXene, and simultaneously stabilize its layered structure during the charging/discharging process. The phase transformation from V_2_C to amorphous vanadium oxide (VO*_x_*) raises the valence states of the outermost V atoms without destroying the layered structure of the internal V_2_C. Thus, the aqueous ZIBs based on the resulting VO*_x_*/Mn–V_2_C cathode exhibit a high specific capacity of 530 mA h g^−1^ at the current density of 0.1 A g^−1^ as well as high energy density of 415 W h kg^−1^. More importantly, the reaction mechanism of the VO*_x_*/Mn–V_2_C electrode is investigated in detail and through multiple analytical methods to explore the electrochemical kinetics and Zn‐ion storage during the charge/discharge process. This study opens an effective avenue for the rational fabrication of electrode materials with high energy density and long cycling life for aqueous ZIBs.

## 2 Results and Discussion

**Figure 1:**
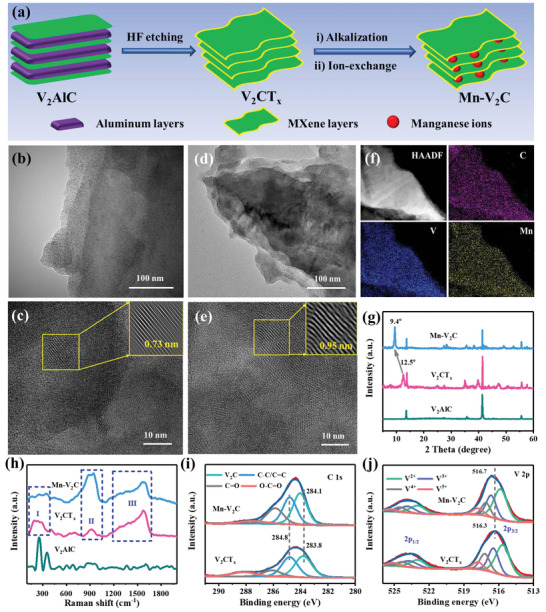
a) Schematic illustration of the synthesis of Mn–V_2_C. TEM and HRTEM images of b,c) V_2_CT*_x_* and d,e) Mn–V_2_C. f) HAADF‐STEM image and the corresponding element mappings of Mn–V_2_C. g) XRD patterns and h) Raman spectrum of V_2_AlC, V_2_CT*_x_*, and Mn–V_2_C. i) High‐resolution C 1s XPS spectra and j) high‐resolution V 2p XPS spectra for V_2_CT*_x_* and Mn–V_2_C.

The synthesis of V_2_CT*_x_* MXene with manganese preintercalation (Mn–V_2_C) is shown in **Figure** **1**a. V_2_CT*_x_* MXene was first obtained from V_2_AlC MAX by HF etching the Al layers. Subsequently, Mn–V_2_C was obtained by treatment of V_2_CT*_x_* with alkali (labeled as K–V_2_C) and orderly ion‐exchange strategies using potassium hydroxide (KOH) and manganese acetate aqueous solution during a liquid‐phase immersion process, respectively (the details are described in the Experimental Section, Supporting Information). The morphological evolutions of the resulting materials throughout the synthesis process were monitored by scanning electron microscopy (SEM). As shown in Figure S1a,b (Supporting Information), SEM images of V_2_AlC MAX show the typical morphology of the ceramic phase,^[^ ^45^ ^]^ which displays a rough surface and the typical densely layer‐stacking structure with micrometer size. V_2_AlC MAX can be exfoliated into V_2_CT*_x_* MXene with an accordion‐like multilayer nanostructure during the HF‐etching process (Figure S1c,d, Supporting Information), which is similar to that of the reported MXenes.^[^ ^46^, ^47^ ^]^ The absence of the Al atom layer leads to the uniform sectional clearance of V_2_CT*_x_*, which is further corroborated by the obviously decreased content of Al observed in the energy dispersive spectroscopy (EDS) spectra (Figure S2, Supporting Information). After intercalation of metal cations, the SEM image of Mn–V_2_C shows no obvious layered strips when compared with V_2_CT*_x_* MXene (Figure S1e,f, Supporting Information), which is mainly ascribed to the intercalation of Mn^2+^ ions into the interlamination of the V_2_CT*_x_* matrix.^[^ ^45^ ^]^

The fine morphological and structural features of V_2_CT*_x_* and Mn–V_2_C were further confirmed by transmission electron microscopy (TEM) and high‐resolution TEM (HRTEM). As shown in Figure 1b, the TEM images show that V_2_CT*_x_* offers stacking morphology to the multilayer nanosheets, in agreement with the SEM results. The interplanar spacing of V_2_CT*_x_*, observed from the HRTEM image is about 0.73 nm (Figure 1c). Similarly, the TEM image of Mn–V_2_C also shows a multilayer nanosheet stacking morphology (Figure 1d). However, the interplanar spacing of Mn–V_2_C is increased to 0.95 nm (Figure 1e), and this could effectively decrease the interfacial barrier for ion transfer and lead to an enhancement of the reaction kinetics. The high‐annular dark‐field scanning TEM (HAADF‐STEM) image and the corresponding elemental mapping confirm the uniform distributions of the elements C, V, and Mn in Mn–V_2_C (Figure 1f), indicating the successful introduction of Mn^2+^ ion into the V_2_CT*_x_* framework. The amount of Mn ion is about 5.04 wt% in the Mn–V_2_C samples and the content of K is substantially decreased from 5.37 wt% in the K–V_2_C to 1.12 wt% according to the EDS spectra (Figure S3, Supporting Information), further indicating the success of the ion‐exchange process.

A typical X‐ray diffraction (XRD) pattern of V_2_AlC MAX shows the distinct diffraction peaks at 13.5° and 41.3° (Figure 1g), which are indexed to the (002) and (103) planes of V_2_AlC, respectively (JCPDS No. 29‐0101).^[^ ^41^ ^]^ After etching with HF, the characteristic peaks of V_2_AlC MAX at 13.5° and 41.3° were significantly weakened, and this is attributed to the extensive removal of Al during etching process. Meanwhile, a new broad peak appeared at 12.5° (Figure 1g), which could be assigned to the (002) plane of V_2_CT*_x_* MXene, demonstrating an extended interlayer spacing of 0.73 nm in comparison with that for V_2_AlC MAX (0.66 nm),^[^ ^45^, ^48^ ^]^ calculated according to Bragg's Law. After the alkalization by KOH, it was observed that the (002) peak shifts to a lower angle of 9.3° (Figure S4a, Supporting Information), indicative of the increased interplanar spacing of 0.96 nm resulted from the intercalation of K^+^. Subsequent replacement of K^+^ ions with Mn^2+^ ions gives a Mn–V_2_C sample showing only a minor increase of 9.4° in the (002) peak when compared with K–V_2_C (Figure 1g and Figure S4b (Supporting Information)). This corresponds to the decreased interlayer spacing of 0.95 nm, which can also be observed in the HRTEM image of Mn–V_2_C. This change is mainly ascribed to the stronger electrostatic interactions of Mn^2+^ ions than K^+^ ions, which paves the way to the successful replacement of K^+^ ions by Mn^2+^ ions.

Raman spectra were recorded in order to investigate the structure and phase transformation from V_2_AlC to V_2_CT*_x_* and finally, to Mn–V_2_C. As shown in Figure 1h, the major peaks of V_2_AlC at 254 and 354 cm^−1^ essentially disappeared from the spectrum of V_2_CT*_x_*, and new broad peaks I and II appeared at 250 and 914 cm^−1^, corresponding to the V—C vibrations.^[^ ^41^, ^48^, ^49^, ^50^ ^]^ In addition, a broad peak III appeared in the range of 1107–1713 cm^−1^, and could be attributed to D and G bands of the layered structures,^[^ ^51^ ^]^ further confirming the successful conversion of V_2_AlC to V_2_CT*_x_*. In the spectrum of Mn–V_2_C, peak I becomes broader but weaker, and peak II downshifts and becomes stronger in comparison with those in V_2_CT*_x_*. These effects are ascribed to the breakup of partial V—V bonds and the formation in their place of a V—O—Mn bond,^[^ ^41^, ^50^, ^51^ ^]^ indicating that the Mn atoms in Mn–V_2_C had been successfully intercalated into V_2_C by forming chemical bonds between V and Mn atoms.^[^ ^25^, ^45^ ^]^ This formation of V—O—Mn bonds in Mn–V_2_C is expected to stable the V_2_C structure during the charging/discharging processes. The electrical conductivities of Mn–V_2_C and V_2_CT*_x_* were examined by the four‐probe method on pressed pellets. They are as high as 1570 ± 50 and 1610 ± 50 S cm^−1^, respectively, which indicates that the high conductivity of MXene is not destroyed during the Mn‐ion preintercalation process.

The interactions between the Mn, C, and V atoms and the chemical structures of V_2_CT*_x_* and Mn–V_2_C were further studied by the X‐ray photoelectron spectroscopy (XPS). As shown in Figure S5 (Supporting Information), the XPS spectrum of V_2_CT*_x_* shows four peaks at 284, 516, 530, and 688 eV, which are assigned to C 1s, V 2p, O 1s, and F 1s, respectively. However, the XPS spectrum of Mn–V_2_C has two additional peaks at 642 and 653 eV, which correspond to Mn 2p_3/2_ and Mn 2p_1/2_, respectively. The high‐resolution C 1s XPS spectrum for V_2_CT*_x_* shows four peaks at 283.8, 284.8, 284.1, and 288.2 eV, which are assigned to V_2_C, C—C/C=C, C=O, and O—C=O, respectively (Figure 1i). The C 1s XPS spectrum of Mn–V_2_C also contains four peaks, for V_2_C, C—C/C=C, C=O, and O—C=O.^[^ ^15^, ^45^ ^]^ It is worth noting that the V_2_C peaks for Mn–V_2_C were upshifted from 283.8 to 284.1 eV when compared to that of V_2_CT*_x_*, and this is attributed to the electronic interactions of the intercalated Mn atoms with V_2_C.^[^ ^52^ ^]^ The V 2p XPS spectra for V_2_CT*_x_* and Mn–V_2_C were deconvoluted into four pairs of doublets (Figure 1j). The doublets at 515.9/523.1, 516.7/523.9, 517.1/524.6, and 517.8/525.5 eV for Mn–V_2_C are assigned to the V^2+^, V^3+^, V^4+^, and V^5+^ states, respectively,^[^ ^42^ ^]^ and the peak intensities of V^2+^ and V^3+^ are relatively higher than those of V^4+^ and V^5+^, indicating that low valence vanadium is dominant in pristine Mn–V_2_C. In addition, the main V 2p peak of Mn–V_2_C shifts toward higher binding energy when compared to that of V_2_CT*_x_*, suggesting that the intercalation of Mn is responsible for the electron exchange among Mn atoms and V_2_C,^[^ ^52^ ^]^ which further validates the existence of chemical binding between Mn and V_2_C. The separation between the two peaks in the Mn 3s orbitals is helpful in the determination of the oxidation state of Mn in manganese oxides. Herein, a separation energy of 6.3 eV for the Mn 3s doublet of Mn–V_2_C confirms the oxidation state of Mn^2+^ (Figure S6a, Supporting Information).^[^ ^7^, ^53^ ^]^ The high‐resolution Mn 2p spectrum displays two peaks at 641.2 eV for Mn 2p_3/2_ and 653.3 eV for Mn 2p_1/2_ (Figure S6b, Supporting Information), which is consistent with those reported for Mn—O. A spin‐energy separation of 12.1 eV between these two peaks further manifests the presence of the Mn—O phase,^[^ ^54^, ^55^, ^56^, ^57^ ^]^ which could be ascribed to the formation of Mn—O—V species among the Mn–V_2_C. Notably, the intercalated Mn could maintain the structure of Mn–V_2_C with accelerated Zn^2+^ transport upon long‐term cycling.^[^ ^58^ ^]^

**Figure 2:**
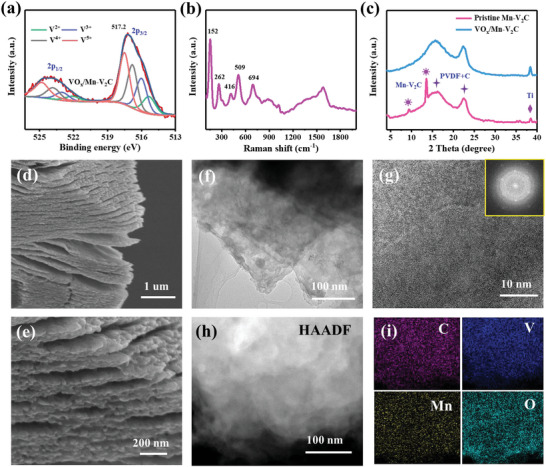
a) High‐resolution V 2p XPS spectra, b) Raman spectrum, and c) XRD pattern for VO*_x_*/Mn–V_2_C. d–f) SEM and TEM images of VO*_x_*/Mn–V_2_C. g) HRTEM image and selected area electron diffraction (SAED) pattern (inset) of VO*_x_*/Mn–V_2_C. h,i) HAADF‐STEM image and the corresponding element mappings of VO*_x_*/Mn–V_2_C.

To explore the superiority of the as‐prepared Mn–V_2_C as a cathode in aqueous ZIBs, the CR2032 coin‐type cells were assembled using zinc foil as the anode and a 2 m ZnSO_4_ aqueous solution as the electrolyte. Owing to the low valence state of V atoms and insufficient active sites in freshly prepared Mn–V_2_C, the Mn–V_2_C electrode shows low initial discharge capacity (Figure S7a, Supporting Information). In order to raise the valence state of V atoms in Mn–V_2_C, an in situ electrochemical oxidation strategy was developed to oxidize the outermost V atoms in the composite electrodes, when Mn–V_2_C electrodes were initially charged up to 1.6 V at 100 mA g^−1^ (Figure S7b, Supporting Information). The valence change of V during charging process was studied in detail by XPS analysis, Raman spectroscopy, and XRD patterns. In the V 2p spectrum of pristine Mn–V_2_C, the V^2+^ and V^3+^ ions are the dominant species relative to V^3+^ and V^4+^ ions (Figure 1j). However, after the first charging process, the V^2+^ and V^3+^ peaks in the activated Mn–V_2_C cathodes gradually weaken, while the peak intensities of V^4+^ and V^5+^ clearly increase (**Figure** **2**a), and meanwhile the main peak of V 2p_3/2_ upshifts toward higher binding energy at 517.2 eV. In addition, Raman spectra also show peaks at 152, 262, 416, 509, and 694 cm^−1^ (Figure 2b), which are exclusively indexed to vanadium oxide.^[^ ^11^, ^34^ ^]^ The broad peaks in the range of 1107–1713 cm^−1^ in the Raman spectra are still present, indicating that the layered structure was not destroyed during the electrochemical oxidization process. However, no typical peaks of vanadium oxide were detected in the XRD pattern (Figure 2c), and the peaks of Mn–V_2_C also become ill‐defined after charging. These results suggest that the in situ formation of amorphous vanadium oxide occurs on the surface of pristine Mn–V_2_C during the first charge process.^[^ ^11^, ^34^ ^]^ These generated high‐valence V species are identified as VO*_x_*, and the activated Mn–V_2_C cathodes were marked as VO*_x_*/Mn–V_2_C.

SEM images show that VO*_x_*/Mn–V_2_C still possesses the accordion‐like layer structure of pristine Mn–V_2_C, and emerging VO*_x_* nanoparticles were observed on the surface of this accordion‐like layer structure morphology (Figure 2d,e), which agrees with the Raman spectra. TEM images further reveal that VO*_x_* mainly exists on the outer surface of Mn–V_2_C, while the multilayered structure is clearly observed (Figure 2f). HRTEM images show no distinct lattice spacings of VO*_x_* (Figure 2g), while selected area electron diffraction (SAED) results display concentric diffraction rings with scattered diffraction spots (Figure 2g, inset), confirming that amorphous VO*_x_* components cover on the surface of inner residual Mn–V_2_C.^[^ ^34^, ^59^ ^]^ The HAADF‐STEM image and the corresponding elemental mappings confirm the homogeneous distribution of Mn, V, C, and O elements among VO*_x_*/Mn–V_2_C (Figure 2i), which also supports the uniform growth of VO*_x_* layer on the Mn–V_2_C surface after the in situ oxidation, and the intentional preservation of the internal conductive 2D multilayers of Mn–V_2_C. Therefore, the electrochemical induction results in the oxidation of the outermost V atoms on Mn–V_2_C to the higher valence VO*_x_*. The VO*_x_*/Mn–V_2_C heterostructure combines the outer layer high‐valence VO*_x_* and the inner conductive Mn–V_2_C, which would equip the VO*_x_*/Mn–V_2_C composite cathode with both high and rapid Zn‐ion storage capability, since multielectron redox reactions could proceed rapidly and extensively. Similarly, V_2_CT*_x_* cathode was performed under the same electrochemical oxidation process, resulting in the in situ formation of VO*_x_*/V_2_CT*_x_* composite electrode (Figure S8, Supporting Information).

**Figure 3:**
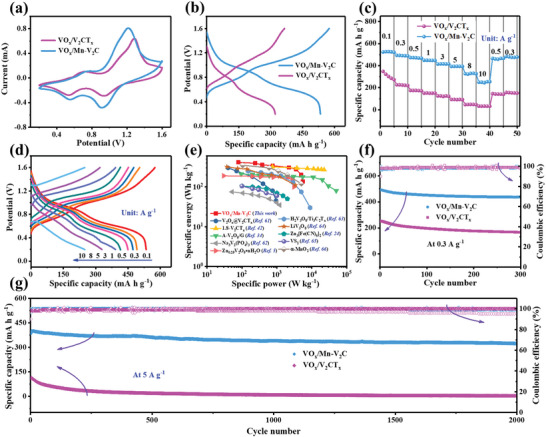
a) CV curves at scan rate of 0.1 mV s^−1^ for VO*_x_*/Mn–V_2_C and VO*_x_*/V_2_CT*_x_*. b) GCD curves of VO*_x_*/Mn–V_2_C and VO*_x_*/V_2_CT*_x_* at 100 mA g^−1^. c) Rate performance of VO*_x_*/Mn–V_2_C and VO*_x_*/V_2_CT*_x_*. d) GCD profiles of VO*_x_*/Mn–V_2_C at different rates. e) Ragone plot of this work compared with other reported cathodes for aqueous zinc‐ion battery. f) Cycling performance of VO*_x_*/Mn–V_2_C and VO*_x_*/V_2_CT*_x_* at 0.3 A g^−1^. g) Cycling performance of VO*_x_*/Mn–V_2_C and VO*_x_*/V_2_CT*_x_* at 5 A g^−1^.

Figure S9 (Supporting Information) shows cyclic voltammetry (CV) curves of activated VO*_x_*/Mn–V_2_C and VO*_x_*/V_2_CT*_x_* electrodes recorded for the first four cycles at a scan rate of 0.1 mV s^−1^. They both show two pairs of redox peaks. The redox peaks at 1.20/0.91 and 0.74/0.54 V for VO*_x_*/Mn–V_2_C are corresponding to the valence changes of vanadium from V^5+^ to V^4+^ and V^4+^ to V^3+^, respectively, indicative of a multistep Zn^2+^ insertion/extraction reaction process.^[^ ^44^, ^60^ ^]^ This similar phenomenon has also been observed in the reported vanadium‐based oxide cathode materials. Moreover, the shapes and positions of these redox peaks process a good reproducibility with almost overlapped during the first four cycles, indicating the high reversibility and excellent stability of the VO*_x_*/Mn–V_2_C electrodes. Apparently, the VO*_x_*/Mn–V_2_C electrode exhibits stronger current density and a larger enclosed area of CV curves than that in VO*_x_*/V_2_CT*_x_* electrode (**Figure** **3**a), indicative of its accelerated electronic/ionic diffusivity and enhanced capacity. Galvanostatic charge‐discharge (GCD) measurements were further conducted to explore the electrochemical performances of the VO*_x_*/Mn–V_2_C and VO*_x_*/V_2_CT*_x_* electrodes (Figure 3b). The VO*_x_*/Mn–V_2_C electrode delivers the initial discharge capacity of 530 mA h g^−1^ at 100 mA g^−1^, clearly higher than VO*_x_*/V_2_CT*_x_* (322 mA h g^−1^). In addition, the GCD profiles of both electrodes display two pairs of plateaus during discharging/charging, which is in good agreement with the CV results.

The rate performance was measured by increasing the current density every five cycles from 0.1 to 10 A g^−1^ with a potential range from 0.2 to 1.6 V. The VO*_x_*/Mn–V_2_C electrode displays much higher discharge capacity and rate performance than VO*_x_*/V_2_CT*_x_* at various current densities (Figure 3c). As the rate increased, the average values of 525, 490, 471, 448, 415, and 393 mA h g^−1^ were measured as the specific discharge capacity at 0.1, 0.3, 0.5, 1, 3, and 5 A g^−1^, respectively. Even at the high rate of 8 and 10 A g^−1^, the corresponding reversible capacity could still maintain 323 and 249 mA h g^−1^, respectively, which is obviously high than that of VO*_x_*/V_2_CT*_x_* at the same rate. Furthermore, when the current density decreases back to 0.5 and 0.3 A g^−1^, the specific discharge capacity can still be 465 and 483 mA h g^−1^, respectively, which is close to initial specific discharge capacity. The GCD profiles show that the polarization degree of VO*_x_*/Mn–V_2_C increases slightly with the progressive increase of the rates from 0.1 to 10 A g^−1^ (Figure 3d), but two charge/discharge platforms are clearly distinguishable even at 5 A g^−1^. Ragone plots further display the superior rate capability of the VO*_x_*/Mn–V_2_C cathode (Figure 3e and Table S1 (Supporting Information)). Clearly, the VO*_x_*/Mn–V_2_C batteries exhibit a superior energy density with 415 W h kg^−1^, and a remarkable power energy density of 5500 W kg^−1^, which is comparable to that of many reported cathode materials for aqueous ZIBs including the V‐based electrode materials.^[^ ^5^, ^24^, ^34^, ^42^, ^61^, ^62^, ^63^, ^64^, ^65^, ^66^ ^]^ Moreover, we have demonstrated the use of prepared aqueous ZIB samples in lighting up a series of light emitting diode (LED) bulbs (Figure S10, Supporting Information), indicating successful device fabrication realized on the lab scale using VO*_x_*/Mn–V_2_C MXene as a cathode material. These results demonstrate that the Zn^2+^ ions can rapidly migrate into the Mn–V_2_C host lattice in VO*_x_*/Mn–V_2_C composites and a redox reaction of V atoms could take place sufficiently on the surficial VO*_x_*, thus providing high specific capability and a considerable rate performance.

The cycling stability performance of VO*_x_*/Mn–V_2_C and VO*_x_*/V_2_CT*_x_* electrodes were initially evaluated at 0.3 A g^−1^. As shown in Figure 3f, VO*_x_*/Mn–V_2_C exhibits an initial discharge capacity of 492 mA h g^−1^, and the capacity remains above 436 mA h g^−1^ after the 300 cycles. A capacity retention of about 89% was achieved at the final cycle with a Coulombic efficiency (CE) of 99% for up to 300 cycles. The electrochemical activities are inferior to those of VO*_x_*/Mn–V_2_C. The specific capacity of VO*_x_*/V_2_CT*_x_* after the 300 cycles is as low as 167 mA h g^−1^, which is only 66% of the initial capacity. Due to the absence of a preintercalated cations, the layer structure of V_2_CT*_x_* was destroyed and the VO*_x_* formed on the surface of V_2_CT*_x_* could be covered during the cycling process (Figure S11a, Supporting Information), resulting in poor cycling performance. However, VO*_x_*/Mn–V_2_C still maintains its layer structure after cycling (Figure S11b, Supporting Information), which promises the rapid transport of Zn^2+^ and effective utilization of VO*_x_*. Even more interestingly, the VO*_x_*/Mn–V_2_C still maintains the high capacity of 323 mA h g^−1^ and high CE of nearly 100% even after 2000 cycles at a high current density of 5 A g^−1^ (Figure 3g), corresponding to capacity retention of 84%. However, VO*_x_*/V_2_CT*_x_* electrode shows an inferior cycling performance with low capacity retention of only 26% after 2000 cycles (Figure 3g), which is ascribed to the expedited collapse of structure for VO*_x_*/V_2_CT*_x_* at high current density. These results strongly support the structural superiorities of Mn–V_2_C as an advanced cathode material for aqueous ZIBs, which can promote the transfer of Zn^2+^ and inhibit occurrence of irreversible structural damage during the long‐life cycling.

In an extension of this research, we also selected Zn^2+^ and Co^2+^ ions as representatives to verify the universality of metal cation preintercalation into V_2_CT*_x_* and capacity enhancement in aqueous ZIBs. The prepared Co–V_2_C and Zn–V_2_C were electrochemically oxidized in situ into VO*_x_*/Co–V_2_C and VO*_x_*/Zn–V_2_C composites following the same process that was used for Mn–V_2_C. As expected, the CV curves for VO*_x_*/Co–V_2_C and VO*_x_*/Zn–V_2_C both display the two pairs of redox peaks (Figure S12, Supporting Information), similar to those in VO*_x_*/Mn–V_2_C. The discharge capacities of VO*_x_*/Co–V_2_C and VO*_x_*/Zn–V_2_C are 485 and 426 mA h g^−1^ at 100 mA g^−1^, respectively (Figure S13a, Supporting Information), and are obviously higher than that of VO*_x_*/V_2_CT*_x_*. Their rate and cycling performances are superior to those for VO*_x_*/V_2_CT*_x_* (Figure S13b,c, Supporting Information). Therefore, these results demonstrate that the introduction of suitable molecule/ion guests into the interlayers of MXene will improve the electrochemical performance of batteries, as could also be observed in the reported works.^[^ ^41^, ^45^, ^48^, ^67^ ^]^

**Figure 4:**
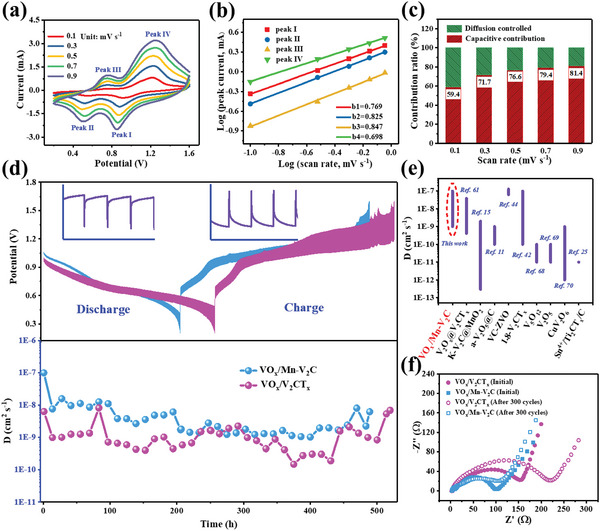
a) CV curves of the VO*_x_*/Mn–V_2_C electrode at different scan rates. b) log *i* versus log *v* plots at selected peak currents. c) The contribution ratio of capacitive and diffusion‐controlled processes in the system with a VO*_x_*/Mn–V_2_C cathode. d) GITT spectra and the corresponding ion‐diffusion coefficient of the VO*_x_*/Mn–V_2_C and VO*_x_*/V_2_CT*_x_* electrodes collected during the third cycle under a current density of 100 mA g^−1^. e) Comparisons between diffusion coefficients of Zn in this work and reported cathodes for aqueous zinc‐ion batteries. f) EIS profiles for VO*_x_*/Mn–V_2_C and VO*_x_*/V_2_CT*_x_* at initial and after 300 cycles states.

To uncover the fundamental origin of the improved rate capacity, electrochemical reaction kinetics of VO*_x_*/Mn–V_2_C were investigated by CV with different scan rates, galvanostatic intermittent titration technique (GITT), and electrochemical impedance spectroscopy (EIS) tests. The CV curves of the VO*_x_*/Mn–V_2_C‐based battery were recorded at scan rates ranging from 0.1 to 0.9 mV s^−1^ (**Figure** **4**a). As the scan rate increases, the cathodic peaks I and II slightly shift during the reduction process to a negative potential, while the anodic peaks III and IV during oxidation process simultaneously move to a positive potential. These peaks, including the area beneath the CV curves, become gradually broader, owing to the increasing polarization effects at higher scan rates, which is a general tendency.^[^ ^61^ ^]^ Moreover, at all the scan rates, the CV curves retain the two pairs of obvious peaks that are in agreement with the better rate performances.

The capacity contributed by the capacitive effect and diffusion effect can be determined by the relationship between the peak current intensity (*i*) and scan rate (*v*) from CV curves, as below^[^ ^44^ ^]^

(1) $$i=av^{b}$$

where the constants of *ɑ* and *b* are empirical parameters, and the value of *b* is defined by the slope of the log *i* versus log *v* graph, as shown in the following equation

(2) logi=blogv+loga

Generally, the values of coefficient *b* vary in a range normally between 0.5 and 1.0. When *b* = 0.5, the electrochemical reaction is a diffusion controlled process, but when the *b* value is close to 1.0, a capacitor‐like process constitutes the main contribution to the electrochemical reaction. The corresponding *b* values of peaks I, II, III, and IV are 0.769, 0.825, 0.847, and 0.698, respectively (Figure 4b), supporting the corresponding redox reactions that are a combination of the capacitive contribution and the ion‐diffusion process.^[^ ^44^ ^]^ To further determine the ratio to the total capacity of the VO*_x_*/Mn–V_2_C electrode, the current response *i* with a fixed potential *V* is regarded as the combination of diffusion‐controlled and surface capacitive behavior, as depicted below

(3) $$i\left(V\right)=k_{1}v+k_{2}v^{1/2}$$

where *k*_1_ *v* and *k*_2_ *v* ^1/2^ define the current contribution of the surface capacitive effect and diffusion‐controlled process, respectively. Based on this equation, the capacitive contribution can be calculated, as shown in Figure 4c. The capacitive contribution is estimated to be 59.4% of the total stored charge at 0.1 mV s^−1^, and with the increasing scan rates, this value gradually increases to 81.4% at 0.9 mV s^−1^, which greatly improves the capacitive ratio of the electrode at higher scan rates. Thus, the short ion‐diffusion length and the high capacitance provided by the rapid electron transfer leads to the superior performance of VO*_x_*/Mn–V_2_C.^[^ ^15^ ^]^

The diffusion kinetics of Zn^2+^ in the VO*_x_*/Mn–V_2_C and VO*_x_*/V_2_CT*_x_* cathodes during cycling was further examined by GITT measurements (Figure 4d). During the charging and discharging processes, the calculated diffusion coefficient (*D*) values of VO*_x_*/Mn–V_2_C are 9.98 × 10^−8^–1.02 × 10^−9^ cm^2^ s^−1^, which is obviously greater than those of VO*_x_*/V_2_CT*_x_* (8.16 × 10^−9^–1.48 × 10^−10^ cm^2^ s^−1^). The *D* values of VO*_x_*/Mn–V_2_C are comparable to those of other reported materials used in aqueous ZIBs (Figure 4e and Table S2 (Supporting Information)),^[^ ^11^, ^15^, ^25^, ^42^, ^44^, ^61^, ^68^, ^69^, ^70^ ^]^ which indicates that VO*_x_*/Mn–V_2_C possesses the rapid diffusion of zinc ions. The higher *D* values can be attributed to preintercalated Mn^2+^ extending the interlayer spacing of the prepared Mn–V_2_C, which provide ordered channels for the rapid diffusion of Zn^2+^. Furthermore, owing to the existence of abundant interfaces between the superficial VO*_x_* and the residual Mn–V_2_C conductive framework, charge redistribution on the interface results in the weakening of electrostatic interactions. This promotes the faster electrochemical kinetics of Zn^2+^ insertion/extraction, and leads to a greater diffusion coefficient of Zn^2+^. The enhanced charge transfer and ionic diffusion ability can be also identified by EIS curves (Figure 4f). The smaller semicircle in VO*_x_*/Mn–V_2_C shows the lower charge transfer resistance, which means the higher conductivity in VO*_x_*/Mn–V_2_C than that of VO*_x_*/V_2_CT*_x_*.^[^ ^71^ ^]^ Moreover, the EIS profiles for VO*_x_*/Mn–V_2_C after 300 cycles exhibit the approximate impedance to that at initial state, whereas VO*_x_*/V_2_CT*_x_* show the obviously increased impedance after 300 cycles, which is ascribed to the expanded interlayer spacing and stable layered structure of MXene benefited from preintercalated Mn ions during the cycling process. Therefore, the synergistic strategies of metal preintercalation and in situ electrochemical oxidization operating on MXene could enhance ion/electron transfer for aqueous zinc‐ion batteries.

**Figure 5:**
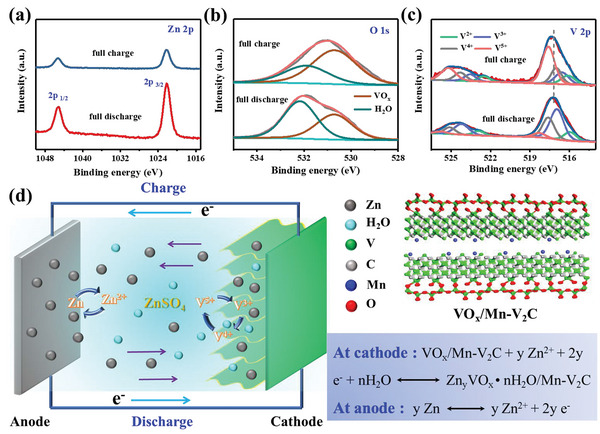
Ex situ XPS spectra of a) Zn 2p, b) O 1s, and c) V 2p for VO*_x_*/Mn–V_2_C cathode in the fully charged and discharged states. d) Schematic and charge storage mechanism of the VO*_x_*/Mn–V_2_C cathode in 2 m ZnSO_4_ electrolyte.

An ex situ XPS analysis was performed to further reveal the insertion/extraction reaction process of the Zn^2+^ ions at the fully discharged and charged states of the VO*_x_*/Mn–V_2_C electrodes by analyzing the differences in the valence state of the major elements, Zn, O, and V. As shown in **Figure** **5**a, the Zn 2p XPS spectra display strong signals at 1022.1 and 1045.1 eV at the fully discharging state. These are attributed to Zn 2p_3/2_ and Zn 2p_1/2_, respectively, demonstrating the existence of the Zn^2+^ that had been successfully intercalated into the host lattice of the VO*_x_*/Mn–V_2_C. Subsequently, while charging to 1.6 V, the intensity of Zn 2p becomes weaker, as the extraction of zinc ions from the VO*_x_*/Mn–V_2_C cathodes proceeds. In the O 1s XPS spectra (Figure 5b), the fitted peak corresponding to H_2_O obviously becomes weaker at fully charged states when compared to fully discharged states, indicating that the Zn^2+^ diffusion process in VO*_x_*/Mn–V_2_C is coupled with the diffusion of H_2_O due to the Zn^2+^ solvation.^[^ ^11^ ^]^

The charging/discharging process would coincide with the valence change of the vanadium in VO*_x_*/Mn–V_2_C (Figure 5c). The V 2p XPS spectra show the presence of V^5+^, V^4+^, V^3+^, and V^2+^ signals, indicating the intrinsic mixed‐valence state of vanadium. At the fully discharged state, V^4+^ and V^3+^ are the dominant species relative to V^5+^. When charged fully to 1.6 V, the V^4+^ and V^3+^ signals decrease and simultaneously, the signal from V^5+^ increases significantly. In addition, the slight upshifting of the main peak positions to high binding energies also demonstrates the increasing average valence states of V from discharging to charging. The above results demonstrate that the Zn||VO*_x_*/Mn–V_2_C batteries display a battery‐type energy release/storage mechanism with the Zn^2+^ reaction as well as the redox of cathode materials.^[^ ^72^ ^]^ Notably, the V^2+^ signals in both states of the V 2p XPS spectra still maintain the similar status. In addition, the concentrations of Mn ions for the cathodes at the fully charged and fully discharged states are 0.93 and 0.89 wt% observed by ex situ EDS spectra (Figure S14, Supporting Information), where these values of concentrations show no obvious change, indicating that Mn ions could stably exist during the energy storage process. As shown Figure S15 (Supporting Information), XRD patterns of VO*_x_*/Mn–V_2_C at fully charged and fully discharged states preserve well and no other impurities occur during the charge/discharge process. This indicates the structural stability of VO*_x_*/Mn–V_2_C cathodes, which is beneficial to the long cycling stability.^[^ ^11^, ^73^ ^]^ In general, the reactions on the electrodes can be tentatively shown in Figure 5d. Coinsertion of Zn^2+^ and H_2_O into the VO*_x_*/Mn–V_2_C host occurs at the cathode and is accompanied by interconversion between V^5+^, V^4+^, and V^3+^ in VO*_x_*, and the corresponding gain or loss of electrons from the zinc metal appears at the anode.

The above observations clearly demonstrate that VO*_x_*/Mn–V_2_C is a promising cathode material in aqueous zinc‐ion batteries. The metal ions that are preintercalated into MXene not only enlarge the interplanar spacing of MXene, but also stabilize the layered structure of MXene, which can promote the charge/electron transfer during the charge/discharge process. The phase transformation from V_2_C to amorphous VO*_x_* induced by in situ electrochemical oxidation could form the heterogeneous interface between residual Mn–V_2_C and the newborn VO*_x_*, which may induce charge redistribution in the interface region and expedite ion or electron transport.^[^ ^52^, ^74^, ^75^ ^]^ Moreover, the in situ phase transformation could also effectively protect us from the toxicity of high valence vanadium during the preparation of materials and assembly of batteries processes. These high valence V atoms from the outermost VO*_x_* participate in reversible interconversion of V species accompanied by the insertion/extraction of Zn^2+^, which promises high capacities of VO*_x_*/Mn–V_2_C. The internal Mn–V_2_C MXene works as the 2D substrate which fully exposes active materials, and meanwhile effectively immobilizes VO*_x_* active species even with a great deal of cycling. These merits enhance electrochemical redox kinetics of VO*_x_*/Mn–V_2_C cooperatively in aqueous zinc‐ion batteries, thus promising high capacities and outstanding cycling stability.

## 3 Conclusion

In summary, we developed dual strategies of metal preintercalation and in situ electrochemical oxidization to deal with the interlayer accumulation and chemical sluggishness of structural elements in MXene used in aqueous ZIBs. The Mn ions preintercalated into the interlaminations could stabilize the layered structure of V_2_CT*_x_*, which in turn could maintain the enlarged interplanar space for frequent transfer of Zn ions during extended cycling. The phase transformation induced by in situ electrochemical oxidization from V_2_C into amorphous VO*_x_* raises the valence of the outermost V under the initial charging process. High‐valence V atoms in VO*_x_* undergo valence interconversion among V^3+^, V^4+^, and V^5+^ accompanied by the insertion/extraction of Zn^2+^ during the discharging/charging process, which leads to a noticeable enhancement of the Zn^2+^ storage capacity. Meanwhile, the inner multilayered structure of V_2_C is maintained intentionally, and provides abundant ordered nanochannels with inherently high electronic/ionic conductivity which supports fast electrochemical reactions. The residual 2D V_2_C also offers a large specific surface for deposition of VO*_x_*, fully exposing active materials and meanwhile effectively immobilizing VO*_x_* active species even after hundreds of cycles. These merits provide the VO*_x_*/Mn–V_2_C cathode with high capacity of 530 mA g^−1^ at 0.1 A g^−1^, and excellent cycling stability of at least 2000 cycles even at 5 A g^−1^. This is conclusive proof of the synergistic effects arising from functionalized strategies operating on MXene. This work offers a satisfactory and low‐toxic vanadium‐based cathode candidate for Zn‐ion storage, and the validated methodology can be used with other MXene materials to enhance ion‐transfer kinetics and electrochemical performances in energy‐storage and ‐conversion systems.

## Conflict of Interest

The authors declare no conflict of interest.

## Supporting information

Supporting Information

## Data Availability

The data that support the findings of this study are available in the supplementary material of this article.
