# Supplementary material for: Dual Strategies of Metal Preintercalation and In Situ Electrochemical Oxidization Operating on MXene for Enhancement of Ion/Electron Transfer and Zinc‐Ion Storage Capacity in Aqueous Zinc‐Ion Batteries
Source: Adv Sci (Weinh). 2023 Jan 16;10(8):2206860. doi: 10.1002/advs.202206860 (PMC10015861; doi:10.1002/advs.202206860)
Supplement: Supplementary file 1 — Supporting Information [file ADVS-10-2206860-s001.pdf]

ADVANCED SCIENCE
Open Access

Supporting Information

for *Adv. Sci.*, DOI 10.1002/advs.202206860

Dual Strategies of Metal Preintercalation and In Situ Electrochemical Oxidization Operating on MXene for Enhancement of Ion/Electron Transfer and Zinc-Ion Storage Capacity in Aqueous Zinc-Ion Batteries

*Zhonglin Li, Yifan Wei, Yongyao Liu, Shuai Yan and Mingyan Wu**

Dual Strategies of Metal Pre-intercalation and *in-situ* Electrochemical Oxidization Operating on MXene for Enhancement of Ions/Electrons Transfer and Zinc Ions Storage Capacity in Aqueous Zinc-Ion Batteries

*Zhonglin Li, Yifan Wei, Yongyao Liu, Shuai Yan and Mingyan Wu**

Experimental Section

Materials

V_2AlC MAX powder was purchased from Foshan Xinxi Technology (Guangdong, China) Co., Ltd. Zinc foil, Titanium foil and Whatman glass filter paper were purchased from Saibo Electrochemical Materials Net. (Tianjin, China) Co., Ltd. N-methyl pyrrolidone (NMP) was purchased from Adamas-beta (Shanghai, China). Acetylene black, poly (vinylidene fluoride) (PVDF) and Zinc sulfate Heptahydrate ($ZnSO_4 \cdot 7H_2O$) were purchased from Aladdin Industrial Corporation (Shanghai, China). All reagents are analytical grade and used as received without further purification.

Structure characterization

Powder X-ray diffraction (XRD) patterns were recorded in the range of 2θ =4-60° on a desktop X-ray diffractometer (RIGAKU-Miniflex 600) with Cu Kα radiation. X-ray photoelectron spectroscopy (XPS) measurements were performed on a Thermos ESCALAB 250 spectrometer using nonmonochromatic Al Kα X-ray as the excitation source and choosing C1s (284.8 eV) as the reference line. Scanning electron microscope (SEM) images were obtained using a JSM-6700Ffield-emission scan electron microscope. Transmission electron microscope (TEM) images were obtained on TECNAI G2F20. Raman spectra were measured by using Raman spectroscopy (Horiba Jobin Yvon) with a 532 nm laser excitation. The electrical conductivities were measured using a linear four-point probe resistivity measurement system (Guangzhou, China) on pellets with 13 mm diameter pressed at 20 M Pa.

Synthesis of V_2CT_x: Multi-layered V_2CT_x MXene was synthesized according to a

modified etching method as we reported previously. [S1] Briefly, 1 g V_2AlC was added into a 100 mL Teflon beaker, followed by dropwise adding 30 mL of 40 wt% HF with continuous magnetic stirring under the argon atmosphere, which was then kept at 35 °C for 72 h. Afterwards, the black precipitate was centrifuged, repeatedly washed with deionized water until neural pH (6-7), and freeze-dried for two days to provide V_2CT_x.

Synthesis of Mn-V_2C: To prepare Mn-intercalated V_2C, the above V_2CT_x powder was first alkalized by immersing in 2 M KOH at 40 °C for 24 hours; then, transferred into 0.2 M manganese acetate at 40 °C for another 48 hours. Mn-V_2C powder was finally obtained after washing and freeze-drying.

Zn-V_2C and Co-V_2C were synthesized using the same procedures except replacing manganese acetate with the zinc acetate and cobaltous acetate, respectively.

Electrochemical characterization

70 wt% active materials (V_2CT_x, Mn-V_2C, Zn-V_2C and Co-V_2C), 20 wt% acetylene black and 10 wt% polyvinylidene difluoride (PVDF) were mixed in 1-methyl-2-pyrrolidinone to form homogeneous slurry. The slurry was pasted onto titanium foil and dried in vacuum oven at 80 ºC for 12 h, subsequent pressing with a roller machine and cutting into circular pieces with diameter of 14 mm generated the desired electrodes. Electrochemical experiments were performed via CR2032 coin-type test cells assembled with zinc foil as the anode in atmosphere. Whatman glass filter paper (GF/B) was used as the separator. The electrolyte was 2 M $ZnSO_4$ dissolved in H_2O. The discharge/charge measurements were conducted at a voltage interval of 0.2 to 1.6 V using a Neware battery test system ((Shen Zhen Neware Technology Co. Ltd). The

cyclic voltammetry (CV) and electrochemical impedance spectroscopy (EIS) measurements were performed on CHI604E electrochemical workstation. The CV scan rate was fixed at 0.1 to 0.9 mV s^{-1}, and the fresh cell was pre-activated by sweeping one cycle before CV measurement. EIS was measured with an applied sinusoidal excitation voltage of 5 mV in the frequency range from 100 kHz to 0.1 Hz.

The Calculation for the diffusion coefficient (D) of Zn^{2+}

Galvanostatic intermittent titration technique (GITT) measurement during the 5th cycle at the current density of 100 A $g^{-1}$is utilized to reveal the Zn^{2+} diffusion coefficient (D_{Zn}) in the VO_x/Mn-V_2C and VO_x/V_2CT_xcathode. During the GITT test, the cell was discharged and charged with the same current density at an interval of 30 second as pulse duration, followed by an open circuit stand (relaxation time) for 30 min to allow the cell voltage to recover its steady-state value. The technique was repeated until the cut-off voltages of the battery operation (1.6 or 0.2 V).

By virtue of the linear relationship of the voltage variation E and $\tau^{1/2}$, the D_{Zn} can be determined based on the following equation: [S2, S3]

$$D_{Zn} = \frac{4L^2}{\pi\tau}(\frac{\Delta E_s}{\Delta E_t})^2$$

where τ is the duration of the current pulse (s); L is the Zn^{2+} ion diffusion length (cm) which is equal to the thickness of electrode for compact electrodes; ΔE_s is the voltage change between two adjacent equilibrium states; and ΔE_τ is the voltage change induced by the galvanostatic charge/discharge.

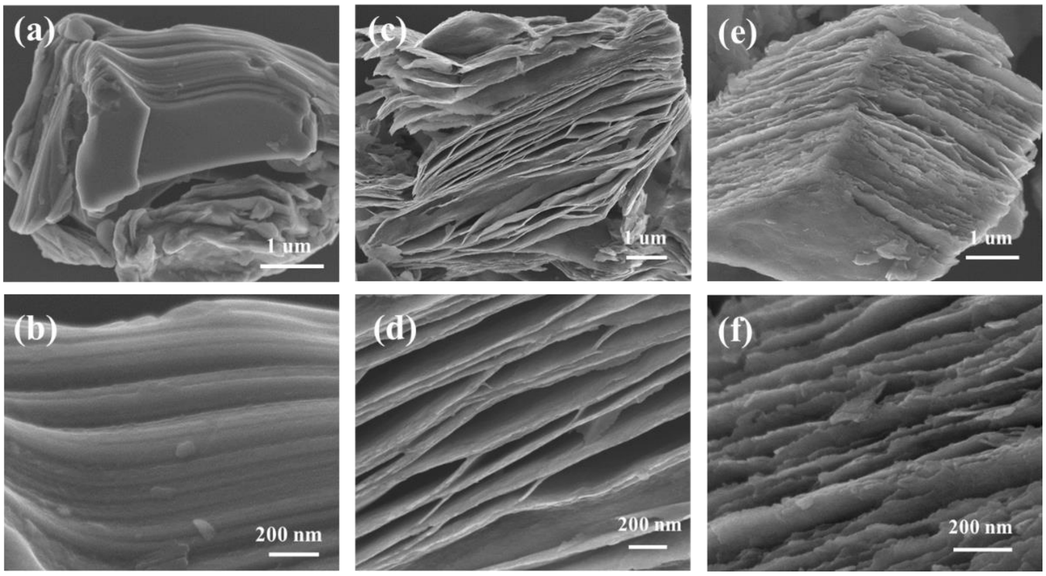

Figure S1. SEM images for (a, b) V_2AlC, (c, d) V_2CT_x and (e, f) Mn-V_2C.

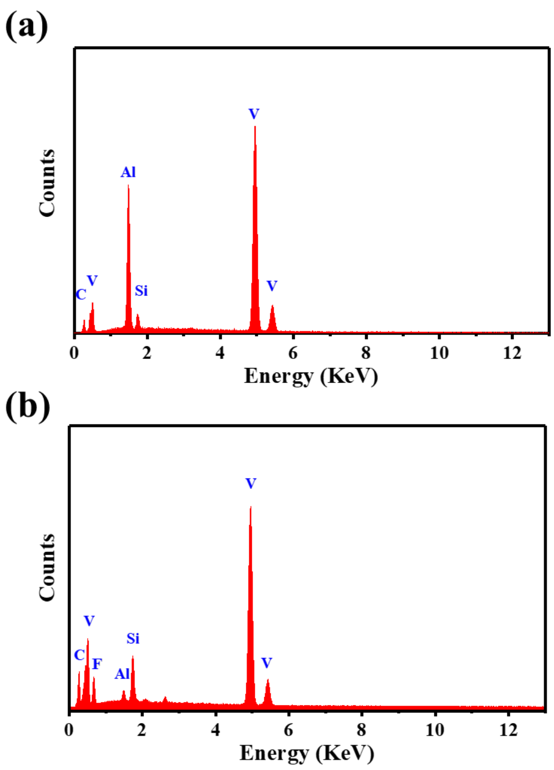

(a)

Elements	Weight (%)
C	10.81
V	67.37
Al	19.65
Other	2.17
Total	100

(b)

Elements	Weight (%)
C	18.05
V	49.50
Al	1.68
F	16.97
Other	13.8
Total	100

Figure S2. Energy dispersive spectroscopy (EDS) spectra of (a) V_2AlC and (b) V_2CT_x.

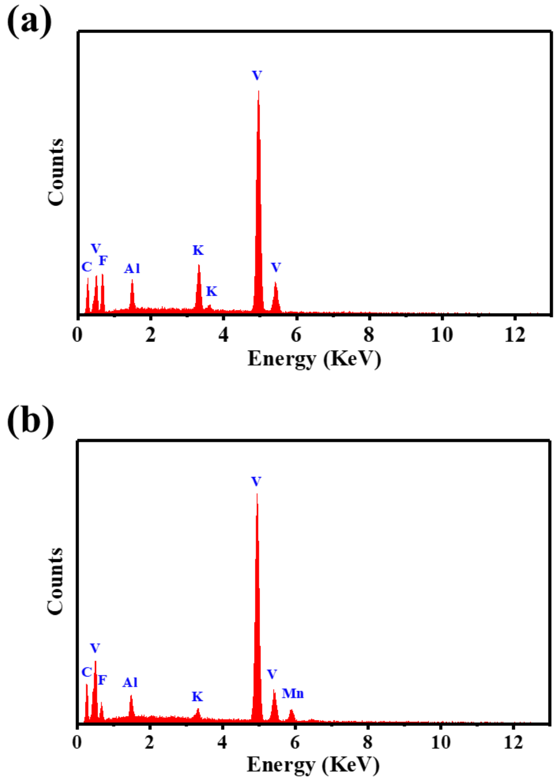

(a)

Elements	Weight (%)
C	12.24
V	56.31
K	5.37
Other	26.08
Total	100

(b)

Elements	Weight (%)
C	20.05
V	57.93
K	1.12
Mn	5.04
Other	15.86
Total	100

Figure S3. EDS spectra of (a) K-V_2C and (b) Mn-V_2C.

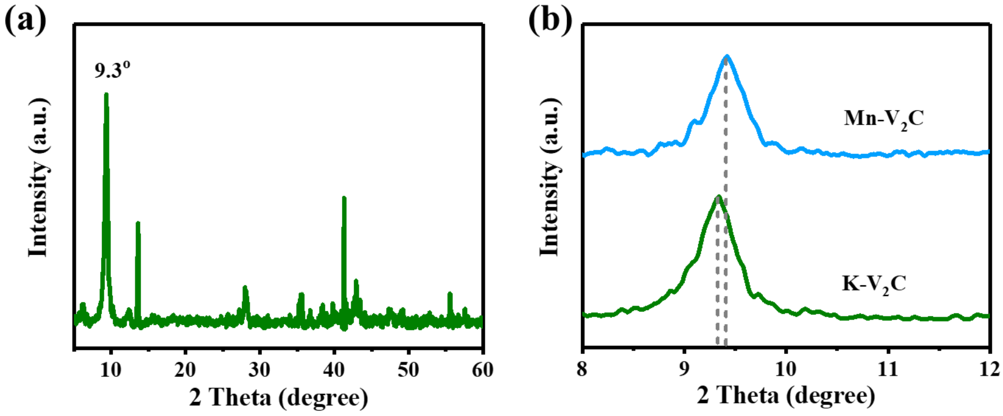

Figure S4. (a) XRD patterns of K-V_2C. (b) The enlarged XRD patterns of K-V_2C and Mn-V_2C.

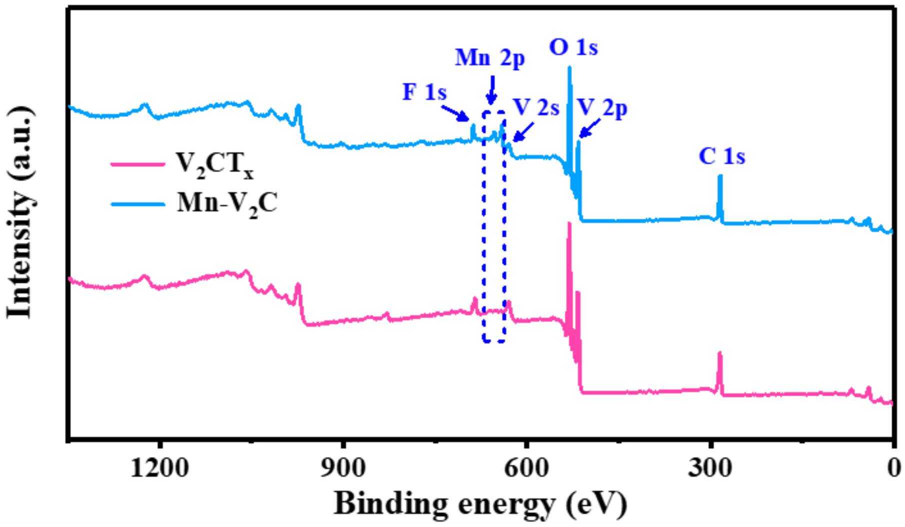

Figure S5. XPS survey spectra of V_2CT_x and Mn-V_2C.

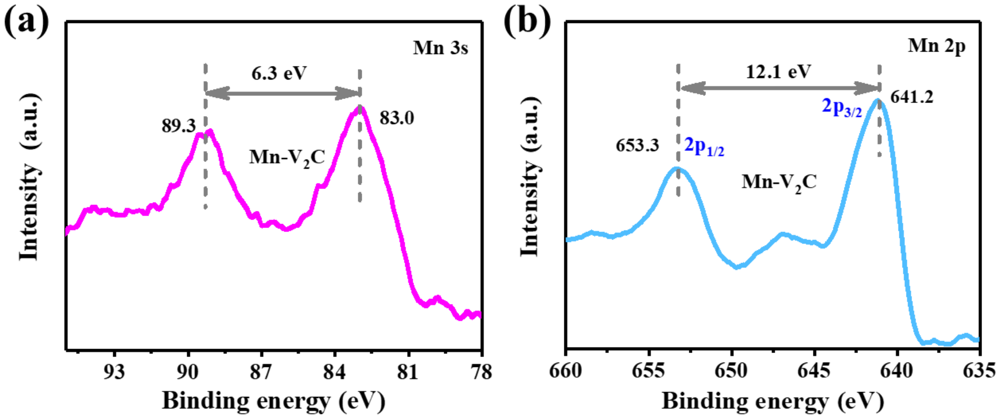

Figure S6. High-resolution (a) Mn 3s and (b) Mn 2p XPS spectra for Mn-V_2C.

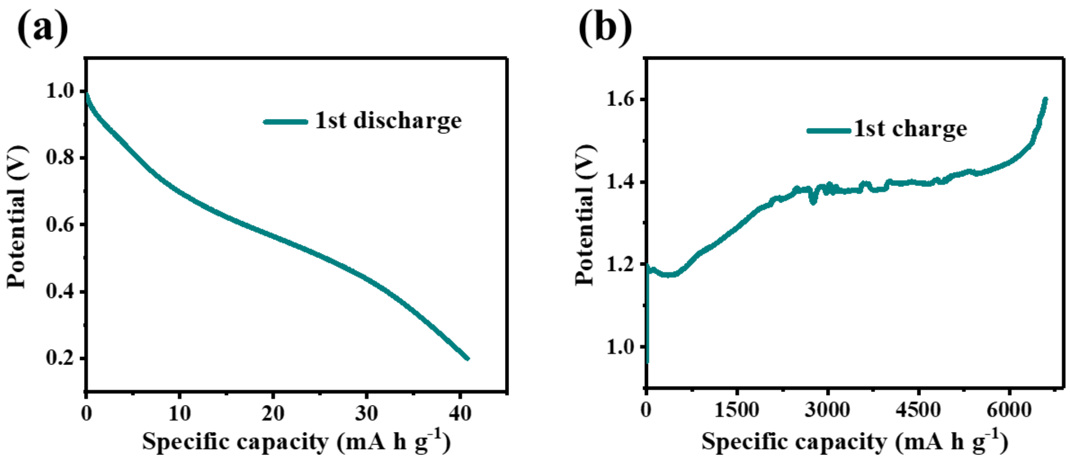

Figure S7. (a) The first discharge curve of Mn-V_2C at a current density of 100 mA g^{-1}. (b) The in-situ electrochemical oxidation curve of Mn-V_2C at a current density of 100 mA g^{-1}.

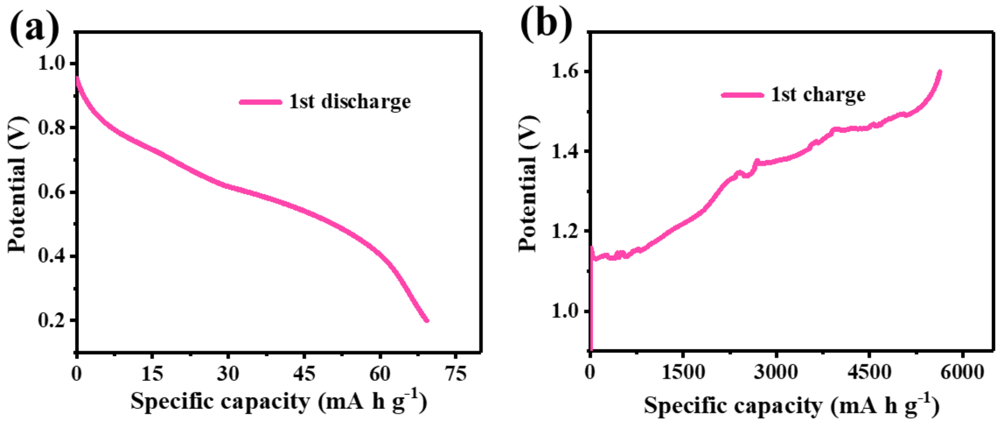

Figure S8. (a) The first discharge curve of V_2CT_x at a current density of 0.1 A g^{-1}. (b) The in-situ electrochemical oxidation curve of V_2CT_x at a current density of 0.1 A g^{-1}.

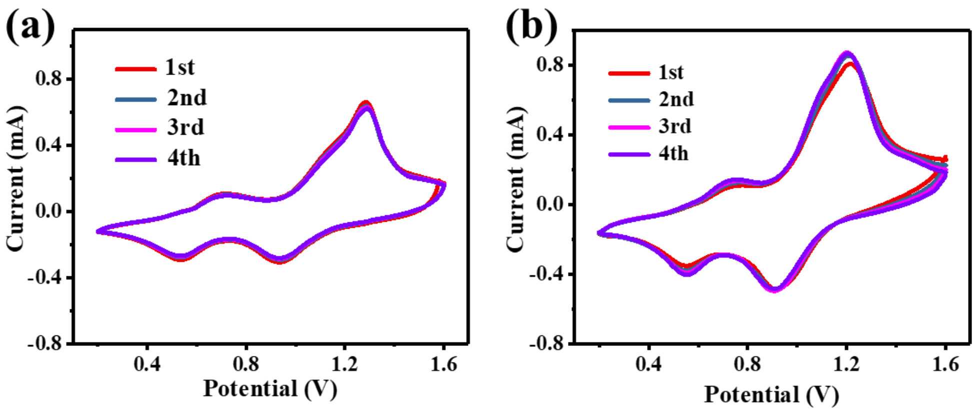

Figure S9.CV curves in the initial four cycles for (a) VO_x/V_2CT_x and (b) VO_x/Mn-V_2C.

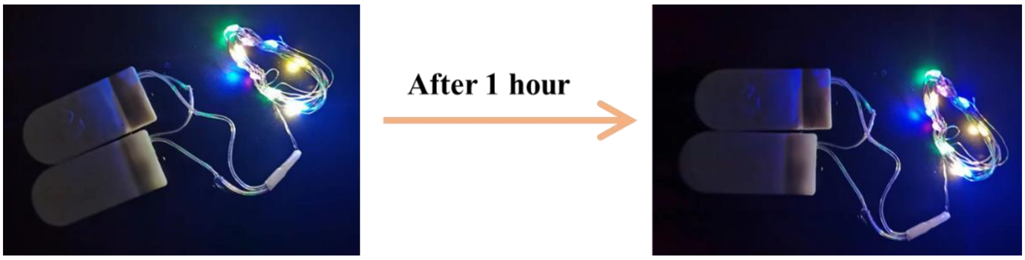

Figure S10. The digital photos show that four ZIBs coin-cell pack in series lights up 10 green indicators of LED modules (circuit diagram is indicated by red arrow) for 1 h.

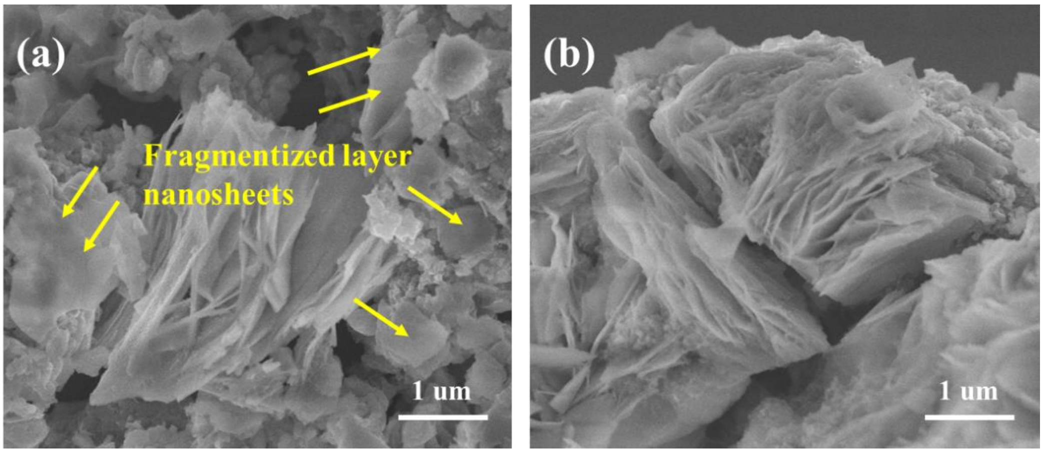

Figure S11. SEM image of (a) VO_x/V_2CT_x and (b) VO_x/Mn-V_2C after cycling.

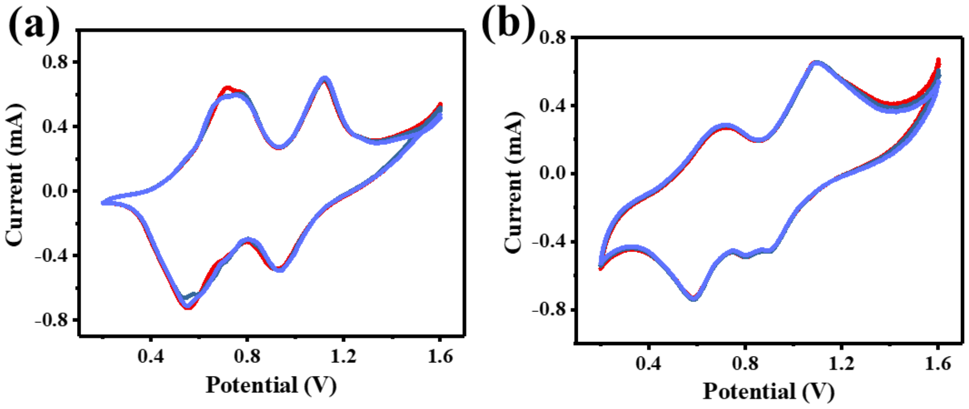

Figure S12. CV curves s for (a) VO_x/Zn-V_2C and (b) VO_x/Co-V_2C.

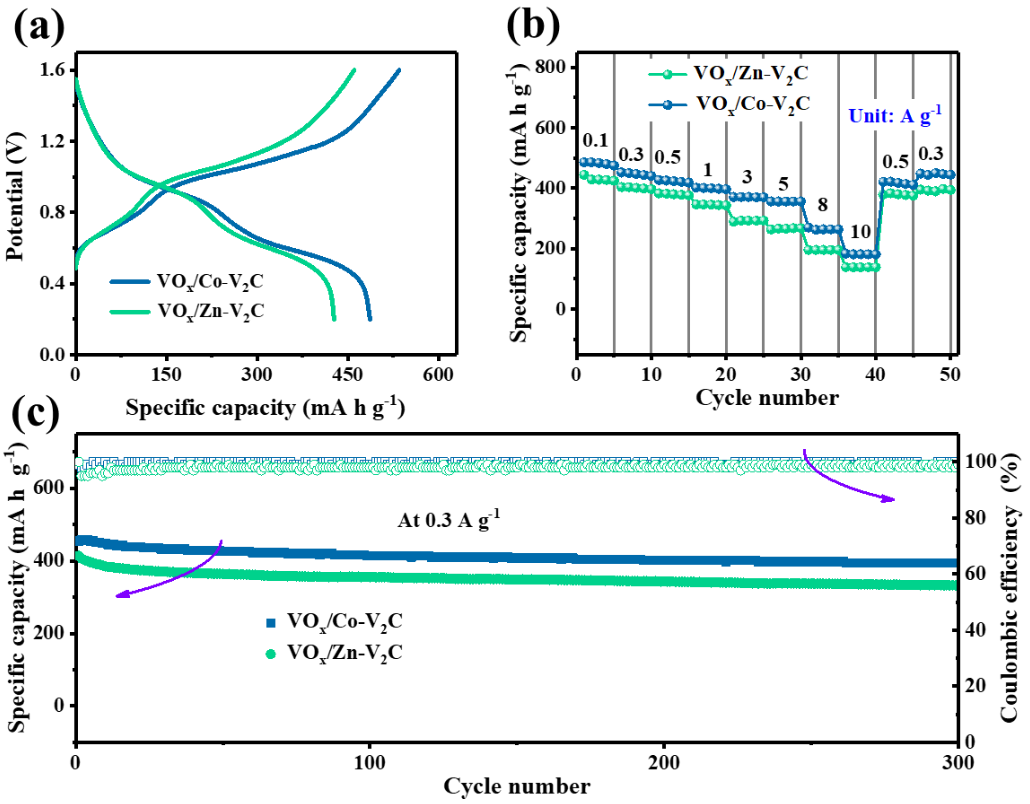

Figure S13. (a) GCD curves of VO_x/Zn-V_2C and VO_x/Co-V_2C at 100 mA g^{-1}. (b) Rate performance for VO_x/Zn-V_2C and VO_x/Co-V_2C. (c) Cycling performance of VO_x/Zn-V_2C and VO_x/Co-V_2C at 0.3 A g^{-1}.

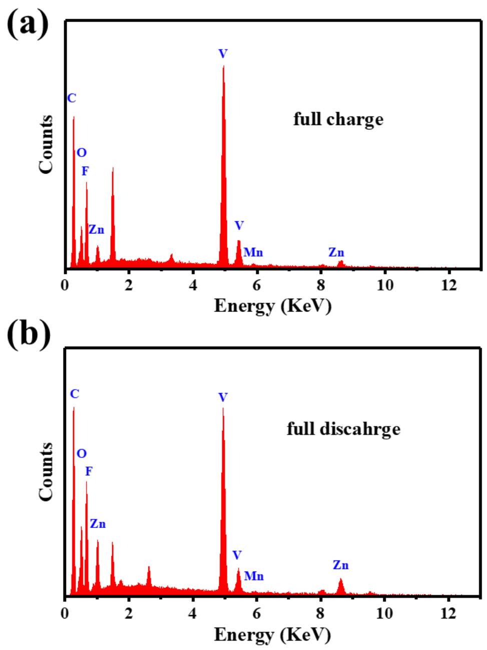

Elements	Weight (%)
V	29.87
Zn	3.32
Mn	0.93
Other	65.88
Total	100

Elements	Weight (%)
V	31.24
Zn	13.65
Mn	0.89
Other	54.22
Total	100

Figure S14. *Ex-situ* EDS spectra of VO_x/Mn-V_2C electrode at (a) the fully charged and (b) fully discharged states.

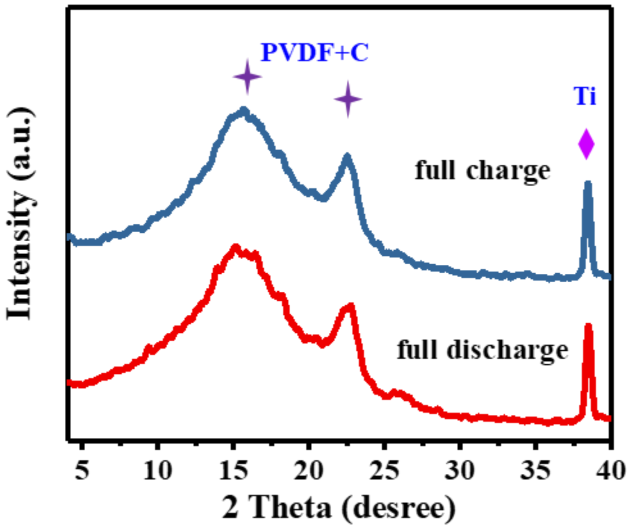

Figure S15. *Ex-situ* XRD patterns of VO_x/Mn-V_2C at the fully charged and discharged states.

Table S1. Comparison of specific energy density and power density of the cathodes between VO_x/Mn-V_2C and the reported other materials.

**Cathode materials**	**Uppermost specific energy density (W h kg-1)**	**Uppermost specific power density (W kg^{-1})**	**Reference**
**VO_x/Mn-V_2C**	**415**	**5500**	**This work**
V_2O_x@V_2CT_x	300	950	[S3]
1.8-V_2CT_x	318	22500	[S4]
A-V_2O_5/G	210	50000	[S5]
$Na_3V_2(PO_4)_3$	75	120	[S6]
$Zn_{0.25}V_2O_5$•nH_2O	190	3100	[S7]
$H_2V_3O_8$/$Ti_3C_2T_x$	250	9000	[S8]
LiV_3O_8	320	3400	[S9]
$Zn_3[Fe(CN)_6]_2$	105	2000	[S10]
VS_2	105	1500	[S11]
α-MnO_2	350	5000	[S12]

Table S2. Comparison for the calculated diffusion coefficient value of Zn ions of the cathodes between VO_x/Mn-V_2C and the reported other materials.

Cathode materials	Diffusion coefficient value range (D, cm^2 s^{-1})	Reference
**VO_x/Mn-V_2C**	**9.98×10^{-8}~1.02×10^{-9}**	**This work**
V_2O_x@V_2CT_x	3.86×10^{-8}~4.22×10^{-10}	[S3]
K-V_2C@MnO_2	2×10^{-9}~3×10^{-13}	[S13]
a-V_2O_5@C	1×10^{-9}~1×10^{-10}	[S14]
VC-ZVO	1.3×10^{-7}~6.3×10^{-8}	[S15]
1.8-V_2CT_x	1×10^{-7}~1×10^{-10}	[S4]
V_5O_{12}	1×10^{-10}~1×10^{-11}	[S16]
V_2O_5	1×10^{-10}~1×10^{-11}	[S17]
CuV_2O_6	1×10^{-9}~1×10^{-12}	[S18]
Sn^{4+}/Ti_2CT_x/C	1×10^{-11}	[S19]

References

[S1] C. Wang, H. Xie, S. Chen, B. Ge, D. Liu, C. Wu, W. Xu, W. Chu, G. Babu, P. M. Ajayan, L. Song, *Adv. Mater.* **2018**, *30*, 1802525.

[S2] N. Duc Tung, H. T. T. Le, C. Kim, J. Y. Lee, J. G. Fisher, I. D. Kim, C. J. Park, *Energy Environ. Sci.* **2015**, *8*, 3577.

[S3] R. Venkatkarthick, N. Rodthongkum, X. Zhang, S. Wang, P. Pattananuwat, Y. Zhao, R. Liu, J. Qin, *ACS Appl. Energy Mater.* **2020**, *3*, 4677.

[S4] Y. Liu, Y. Jiang, Z. Hu, J. Peng, W. Lai, D. Wu, S. Zuo, J. Zhang, B. Chen, Z. Dai, Y. Yang, Y. Huang, W. Zhang, W. Zhao, W. Zhang, L. Wang, S. Chou, *Adv. Funct. Mater.* **2020**, *31*, 2008033.

[S5] X. Wang, Y. Li, S. Wang, F. Zhou, P. Das, C. Sun, S. Zheng, Z. Wu, *Adv. Energy Mater.* **2020**, *10*, 2000081.

[S6] G. Li, Z. Yang, Y. Jiang, C. Jin, W. Huang, X. Ding, Y. Huang, *Nano Energy* **2016**, *25*, 211.

[S7] D. Kundu, B. D. Adams, V. Duffort, S. H. Vajargah, L. F. Nazar, *Nat. Energy* **2016**, *1*, 16119.

[S8] P. Liang, T. Xu, K. Zhu, Y. Rao, H. Zheng, M. Wu, J. Chen, J. Liu, K. Yan, J. Wang, R. Zhang, *Energy Storage Mater.* **2022**, *50*, 63.

[S9] P. He, M. Yan, X. Liao, Y. Luo, L. Mai, C.-W. Nan, *Energy Storage Mater.* **2020**, *29*, 113.

[S10] L. Zhang, L. Chen, X. Zhou, Z. Liu, *Adv. Energy Mater.* **2015**, *5*, 1400930.

[S11] P. He, M. Yan, G. Zhang, R. Sun, L. Chen, Q. An, L. Mai, *Adv. Energy Mater.*

2017, *7*, 1601920.

[S12] B. Wu, G. Zhang, M. Yan, T. Xiong, P. He, L. He, X. Xu, L. Mai, *Small* **2018**, *14*, 1703850.

[S13] X. Zhu, Z. Cao, W. Wang, H. Li, J. Dong, S. Gao, D. Xu, L. Li, J. Shen, M. Ye, *ACS Nano* **2021**, *15*, 2971.

[S14] S. Deng, Z. Yuan, Z. Tie, C. Wang, L. Song, Z. Niu, *Angew. Chem. Int. Ed.* **2020**, *59*, 22002.

[S15] X. Zhu, W. Wang, Z. Cao, S. Gao, M. O. L. Chee, X. Zhang, P. Dong, P. M. Ajayan, M. Ye, J. Shen, *J. Mater. Chem. A* **2021**, *9*, 17994.

[S16] N. Zhang, M. Jia, Y. Dong, Y. Wang, J. Xu, Y. Liu, L. Jiao, F. Cheng, *Adv. Funct. Mater.* **2019**, *29*, 1807331.

[S17] N. Zhang, Y. Dong, M. Jia, X. Bian, Y. Wang, M. Qiu, J. Xu, Y. Liu, L. Jiao, F. Cheng, *ACS Energy Lett.* **2018**, *3*, 1366.

[S18] Y. Liu, Q. Li, K. Ma, G. Yang, C. Wang, *ACS Nano* **2019**, *13*, 12081.

[S19] X. Li, M. Li, Q. Yang, D. Wang, L. Ma, G. Liang, Z. Huang, B. Dong, Q. Huang, C. Zhi, *Adv. Energy Mater.* **2020**, *10*, 2001394.
